# Genetic Mapping With Allele Dosage Information in Tetraploid *Urochloa decumbens* (Stapf) R. D. Webster Reveals Insights Into Spittlebug (*Notozulia entreriana* Berg) Resistance

**DOI:** 10.3389/fpls.2019.00092

**Published:** 2019-02-21

**Authors:** Rebecca Caroline Ulbricht Ferreira, Letícia Aparecida de Castro Lara, Lucimara Chiari, Sanzio Carvalho Lima Barrios, Cacilda Borges do Valle, José Raul Valério, Fabrícia Zimermann Vilela Torres, Antonio Augusto Franco Garcia, Anete Pereira de Souza

**Affiliations:** ^1^Center for Molecular Biology and Genetic Engineering, University of Campinas, Campinas, Brazil; ^2^Genetics Department, Escola Superior de Agricultura “Luiz de Queiroz”, University of São Paulo, Piracicaba, Brazil; ^3^Embrapa Beef Cattle, Brazilian Agricultural Research Corporation, Campo Grande, Brazil; ^4^Plant Biology Department, Biology Institute, University of Campinas, Campinas, Brazil

**Keywords:** allele dosage, *Brachiaria*, linkage map, polyploidy, signalgrass, SNP, quantitative traits

## Abstract

*Urochloa decumbens* (Stapf) R. D. Webster is one of the most important African forage grasses in Brazilian beef production. Currently available genetic-genomic resources for this species are restricted mainly due to polyploidy and apomixis. Therefore, crucial genomic-molecular studies such as the construction of genetic maps and the mapping of quantitative trait loci (QTLs) are very challenging and consequently affect the advancement of molecular breeding. The objectives of this work were to (i) construct an integrated *U. decumbens* genetic map for a full-sibling progeny using GBS-based markers with allele dosage information, (ii) detect QTLs for spittlebug (*Notozulia entreriana*) resistance, and (iii) seek putative candidate genes involved in defense against biotic stresses. We used the *Setaria viridis* genome a reference to align GBS reads and selected 4,240 high-quality SNP markers with allele dosage information. Of these markers, 1,000 were distributed throughout nine homologous groups with a cumulative map length of 1,335.09 cM and an average marker density of 1.33 cM. We detected QTLs for resistance to spittlebug, an important pasture insect pest, that explained between 4.66 and 6.24% of the phenotypic variation. These QTLs are in regions containing putative candidate genes related to defense against biotic stresses. Because this is the first genetic map with SNP autotetraploid dosage data and QTL detection in *U. decumbens*, it will be useful for future evolutionary studies, genome assembly, and other QTL analyses in *Urochloa* spp. Moreover, the results might facilitate the isolation of spittlebug-related candidate genes and help clarify the mechanism of spittlebug resistance. These approaches will improve selection efficiency and accuracy in *U. decumbens* molecular breeding and shorten the breeding cycle.

## Introduction

Brazilian cultivated pastures are the basis for the production of beef cattle, and they cover extensive areas, most of which are populated by grasses of the genus *Urochloa* (syn. *Brachiaria*) (Jank et al., 2011; Associação Brasileira das Indústrias Exportadoras de Carne [ABIEC], 2016). This genus belongs to the Poaceae family and comprises species of plants distributed in tropical and subtropical regions, mainly in the African continent (Renvoize et al., 1996; Valle et al., 2009).

One of the most widely cultivated species of genus is *Urochloa decumbens* (Stapf) R. D. Webster, also known as signalgrass. This forage grass has relevant agronomic attributes, including exceptional adaptation to poor and acidic soils that are typical of the tropics, leading to good animal performance (Valle et al., 2010). However, the species is susceptible to several types of spittlebug, including *Notozulia entreriana* Berg (Hemiptera: Cercopidae), which is the most damaging pest for tropical pastures (Gusmão et al., 2016).

Spittlebugs reduce biomass production, decrease palatability, and reduce the carrying capacity of pastures (Valério and Nakano, 1988). *N. entreriana* nymphs constantly suck the sap of roots, causing yellowing of the plant, and the saliva of the adults induces phytotoxemia that causes plant death (Valério and Nakano, 1992; Gusmão et al., 2016). Therefore, breeding programs aim to develop new cultivars with reasonable or high resistance to spittlebugs to reduce forage loss. To achieve this advance, it is essential to understand the genetic mechanisms involved in the spittlebug resistance response in forage grass and to identify valuable markers for selection in breeding schemes. Currently, spittlebug resistance in *Urochloa* grasses is thought to be under relatively simple genetic control and is highly heritable (Miles et al., 1995; Miles et al., 2006). Therefore, despite being a trait with little genetic information, spittlebug resistance can probably be easily manipulated in a plant breeding program.

Genetic breeding of *U. decumbens* is recent and has proven challenging because this grass is predominantly tetraploid (2n = 4x = 36) and apomictic, reproducing mainly by facultative apospory (Naumova et al., 1999; Valle et al., 2008). In this scenario, the number of currently available molecular genetic resources is limited. Previous molecular studies have increased knowledge of *U. decumbens* genetics, including the development of sets of microsatellite markers (Ferreira et al., 2016; Souza et al., 2018) and the first transcriptome (Salgado et al., 2017) of the species as well as linkage maps from the interspecific progeny of *U. decumbens* × *U. ruziziensis* (Worthington et al., 2016). Other studies with molecular markers have analyzed the genetic relationships of this species to other species in the *Urochloa* genus (Almeida et al., 2011; Pessoa-Filho et al., 2017; Triviño et al., 2017).

Until recently, it was not possible to perform intraspecific crossings due to the ploidy barriers between apomictic (polyploid) and sexual (diploid) accessions, but this restriction changed when diploid accessions were artificially tetraploidized using colchicine (Simioni and Valle, 2009). This advance allowed the generation of a base population and the exploration of a genetic variability previously conserved by apomixis, favoring new molecular studies, including building genetic linkage maps, which provide useful information about the species.

Genetic maps are essential tools for genetics and genomics research, and they are becoming increasingly common in polyploids species, mainly due to the development of next-generation-sequencing (NGS) technologies and advances in genetic and statistical methods (Garcia et al., 2013). In this study, we utilized the genotyping-by-sequencing (GBS) technique, which enables the discovery of 1000s of SNP at low cost (Elshire et al., 2011; Poland et al., 2012). In addition, robust software tools have been developed for polyploid organisms, including programs that can estimate the allele dosage of SNPs and provide more genetic information to generate linkage maps with high marker densities (Serang et al., 2012; Garcia et al., 2013; Mollinari and Serang, 2015; Bourke et al., 2016, 2018; Gerard et al., 2018).

The mapping process is more complex in polyploid species, mainly because of the larger number of possible genotypes (Ripol et al., 1999; Garcia et al., 2013; Vigna et al., 2016). For example, in autotetraploid species, five possible dosage classes exist: nulliplex (aaaa), simplex (Aaaa), duplex (AAaa), triplex (AAAa), and quadruplex (AAAA). Most polyploid genetic maps have been limited to the use of single-dose markers (SDMs). In this case, either each marker is considered as a single-allele copy from only one of the parents of the cross, with a 1:1 segregation ratio, or the SDMs in both parents segregate in a 3:1 ratio (Wu et al., 1992). This method has been developed and successfully adopted for various forage grass species, such as *Panicum maximum* (Ebina et al., 2005), *Paspalum notatum* (Stein et al., 2007), *Panicum virgatum* (Okada et al., 2010), and *Urochloa humidicola* (Vigna et al., 2016). However, SDMs represent only a partial sample of loci available in the genome, and not all configurations that can be present in the progeny. All possible offspring genotype configurations in an autotetraploid cross were detailed by Hackett et al. (2013) and have been considered in this work. Knowledge of the dosage of an SNP is essential for genetic studies in polyploid species and can significantly increase the information imparted by each locus (Voorrips et al., 2011; Garcia et al., 2013; Bourke et al., 2016, 2018).

High-resolution genetic linkage mapping for polyploid species can identify beneficial trait loci and allow genomics-based breeding programs (Shirasawa et al., 2017). Molecular markers have been widely used to locate quantitative trait loci (QTLs) associated with quantitative resistance to insects in many crop plants (Samayoa et al., 2015; Oki et al., 2017; Vosman et al., 2018); however, genomic loci related to resistance to spittlebugs have not been determined in any plant species. Particularly in *U. decumbens*, the identification of loci involved in this trait is a promising tool for characterizing the genetic architecture and can assist in the design of strategies to be introduced into breeding programs in order to increase the efficiency of the selection processes and accelerate the release of new cultivars (Valle et al., 2009).

This study reports the development and application of GBS for mapping studies in an intraspecific progeny of *U. decumbens*. To the best of our knowledge, there have been no reports of QTL mapping in signalgrass. Our goals were to (i) build a GBS-based integrated genetic map using autotetraploid allele dosage information in a bi-parental progeny, (ii) identify QTLs related to spittlebug resistance on the integrated genetic map, and (iii) search for putative candidate genes that may be involved in spittlebug resistance.

## Materials and Methods

### Plant Material and DNA Extraction

At the Brazilian Agricultural Research Corporation (Embrapa) Beef Cattle (EBC), Campo Grande/MS, an intraspecific cross was made between *U. decumbens* D24/27 (sexual accession tetraploidized by colchicine) and *U. decumbens* cv. Basilisk (tetraploid, apomictic cultivar used as the pollen donor); therefore, both parents were tetraploid. *U. decumbens* cv. Basilisk presents relevant agronomic traits and is therefore an important genotype to the breeding program. The full-sib progeny of 239 F_1_ individuals was analyzed, from which 217 hybrids were identified using SSRs markers obtained from Ferreira et al. (2016).

Leaf samples from each hybrid and both parents were collected, and genomic DNA was extracted using the DNeasy 96 Plant Kit (Qiagen GMbH, Germany). DNA concentrations were determined using a Qubit fluorometer (Invitrogen, Carlsbad, CA, United States).

### GBS Library Construction and Sequencing

The GBS library was prepared at the University of Campinas following the protocol described by Elshire et al. (2011). Samples from both parents of the progeny were replicated five times for sequencing. Each individual within a library was part of a 96-plex reaction. Each DNA sample (300 ng in a volume of 10 μL) was digested with the restriction enzyme NsiI (NEB) to reduce genomic complexity and then ligated to a unique barcoded adapter plus a common adapter. The 96-plex libraries were checked for quality using an Agilent DNA 1000 Kit on an Agilent 2100 Bioanalyzer (Agilent Technologies, Santa Clara, CA, United States). Libraries sequencing was performed as 150-bp single-end reads on the Illumina NextSeq 500 platform. The quality of the resulting sequence data was then evaluated with the NGS QC Toolkit v2.3.3 (Patel and Jain, 2012).

### SNP Calling

SNP discovery and genotype calling were performed using Tassel-GBS pipeline (Glaubitz et al., 2014), which was modified to obtain the original count of the number of reads for each SNP allele (Pereira et al., 2018). Because this pipeline requires a reference genome, and because the *U. decumbens* genome has not yet been sequenced, we aligned the GBS reads using the *Setaria viridis* genome (v 1.0; ∼394.9 Mb arranged in nine chromosomes and 724 scaffolds; diploid forage) as an alternative pseudo-genome. This genome is available from the Phytozome website^1^ (Goodstein et al., 2012), and it sequence data were produced by the US Department of Energy Joint Genome Institute. The Bowtie2 algorithm version 2.1 (Langmead and Salzberg, 2012) was used to align reads against the pseudo-reference with -D and -R parameters defined as 20 and 4, respectively, and with the very-sensitive-local argument.

### Allele Dosage Estimation and Data Filtering

SuperMASSA software (Serang et al., 2012; Pereira et al., 2018) was used to estimate the allele dosage (number of copies of each allele) of each individual. The minimum overall depth considered was 25 reads, and the model used was the F_1_ Population Model. Markers were fitted and filtered to fixed ploidy level of 4.

Additionally, monomorphic SNP markers in the parents and in the progeny were removed, and markers with a maximum of 25% missing data were selected manually using scripts written using R software (R Core Team, 2018).

### Linkage Map

A consensus linkage map was built using the TetraploidSNPMap (TPM, BioSS) software (Hackett et al., 2017), following the methodology described in Hackett et al. (2013, 2014). We used the supposed synteny between *U. decumbens* and *S. viridis* to infer the homologous groups, which were constructed separately using the SNPs identified on each relative *S. viridis* chromosome. Markers with significance of the χ^2^ goodness-of-fit statistic less than 0.001 for simplex and 0.01 for higher segregations were considered distorted and were removed by the TPM software. A total of 2,725 distorted SNPs were excluded from the cluster analyses. The remaining 1,515 non-distorted SNPs were clustered into nine homologous groups using a Chi-square test for independent segregation (Hackett et al., 2013).

Considering the ordination of markers, a two-point analysis was used to calculate the recombination frequency and LOD score for each pair of SNPs in each possible phase by an expectation-maximization (EM) algorithm to maximize the likelihood. Duplicate and near-duplicate SNPs were removed in this step. The recombination frequencies were converted to map distances using Haldane’s mapping function, and a multi-dimensional scaling analysis (MDS) was then performed to calculate the best order for the SNPs in the homology group. After this step, we excluded some SNPs that showed low LOD scores or that were distant from the rest of the group (Hackett et al., 2017). Some phases of the ordered SNPs were inferred by automated analysis in TetraploidSNPMap, and others were manually inferred based on the most likely phases, following the reasoning implemented in software such as OneMap (Margarido et al., 2007).

The genetic map was drawn using MapChart 2.32 (Voorrips, 2002), in which SNP configurations were identified with different colors.

### Phenotypic Evaluation

The antibiosis resistance levels of *U. decumbens* to spittlebugs (*N. entreriana*) was evaluated in greenhouse experiments at Embrapa Beef Cattle, Brazil, according to the methodology described by Lapointe et al. (1992) and Valério et al. (1997). After establishment of seedlings, each individual was infested with five *N. entreriana* spittlebug eggs. The variable evaluated was the percentage of nymphal survival (number of nymphs that reached the adult stage, as a percentage), which is a quantitative trait.

From 2011 to 2013, a total of 12 experiments were conducted; each experiment had a randomized complete block design (RCBD) with different numbers of blocks and treatments. Common treatments were repeated in all experiments and were used as checks in the statistical analysis. A total of 349 individuals were evaluated for spittlebug resistance, of which 157 were hybrids of the mapping population.

In 2011, five RCBD experiments were conducted, with 10 blocks each and a total of 114 individuals evaluated. The best hybrids were selected with respect to spittlebug resistance and included in the experiments conducted in 2013, when seven RCBD experiments were conducted, with four blocks each and a total of 259 individuals evaluated. The common checks for all experiments were *U. decumbens* cv. Basilisk, *U. brizantha* cv. Marandu, *Urochloa* spp. cv. BRS Ipyporã, and *Urochloa* spp. MulatoII. The following statistical linear mixed model was used for the analysis:

$$y_{ijlk}=\mu +b_{j(l)}+p_{l}+a_{k}+t_{ik}+\varepsilon_{ijlk}$$

where y_ijlk_ is the phenotype of the *i*-th individual, at *j*-th block, *l*-th experiment, and *k*-th year; μ is the overall mean; b_j(l)_ is the random effect of the *j*-th block within the *l*-th experiment (b_j(l)_ ∼ N(0,$\sigma_{B}^{2}$)); p_l_ is the random effect of the *l*-th experiment (p_l_ ∼ N(0,σp^2^)); a_k_ is the fixed effect of the *k*-th year; t_ik_ is the random effect of the *i*-th individual in the *k*-th year (t_ik_ ∼ N(0,G)), in which G is the variance-covariance (VCOV) matrix for genetic effects; and 𝜀_ijlk_ is the residual error (𝜀_ijlk_ ∼ N(0,${\hat{\sigma }}_{R}^{2}$)). The G matrix is indexed by two factors (genotype and year) written as the Kronecker product of matrices, G = I_n_ ⊗ G_a_, in which I_n_ is an identity matrix relative to genotype effects and G_a_ is the VCOV matrix relative to year effects. The G_a_ matrix was evaluated considering six different VCOV structures: independent (ID), diagonal (DIAG), compound symmetry (CS), compound symmetry heterogeneous (CS-Het), first-order factor analytic (FA1), and unstructured (US). The selection model was performed considering the Akaike Information Criteria (AIC) (Akaike, 1974) and Schwarz Information Criteria (SIC) (Schwarz, 1978).

Phenotypic analysis was performed using the packages ASReml-R v3 (Butler et al., 2009; Gilmour et al., 2009) and ASRemlPlus (Brien, 2016). Heritability between genotype means was estimated using the index as presented by Cullis et al. (2006) and Piepho and Möhring (2007), where:

$${\hat{\text{H}}}_{\text{C}}^{\text{2}}=1-\frac{PEV}{2\sigma_{G}^{2}}$$

in which PEV is the prediction error variance, and $\sigma_{G}^{2}$ is the genetic variance.

### QTL Mapping

Quantitative trait loci mapping for spittlebug resistance was performed by applying an Interval Mapping (IM) model based on autotetraploid allele dosage information with TetraploidSNPMap software (Hackett et al., 2017). This analysis was performed separately for each homology group using the SNP data for each one of them, the integrated genetic map with phase information and the phenotypic trait data. The interval mapping was fit to a model of additive effects using a weighted regression approach. A significant QTL was declared if the LOD score was above the 95% threshold obtained from a permutation test with 1,000 permutations. From a significant QTL, simple models such as a simplex, duplex or double-simplex QTL models were then tested using the SIC (Schwarz, 1978) to determine if the estimated genotype corresponded to the most likely QTL location. The SIC is calculated in TetraploidSNPMap as follows:

$$\text{SIC}\;=\;-2logL+p\log m_{o},$$

where *L* is the likelihood for the simple model, p is the number of parameters in the simple model, and m_o_ is the number of observations (the 36 genotype means). Models with the lowest value for the SIC were considered the best models (Hackett et al., 2014).

If two closely linked significant QTLs were identified on the same chromosome, TetraploidSNPMap could not estimate the individual QTL LOD and parameter values, yielding data only pertaining to the largest effect.

### Search for Candidate Genes in Detected QTL Regions

We investigated the functional annotation of the candidate genes located close to the QTL regions associated with spittlebug resistance. Using the chromosomal locations of the markers adjacent to QTLs in the *S. viridis* genome as a reference, we aligned the sequences found in these regions (100 kb approximately) with a BLASTX search (e-value cutoff of 1e-0.5) against plants databases using the JBrowse tool^2^. Finally, from this alignment, we selected only genes reported in the literature as associated with resistance/defense against biotic stresses that were present in the transcriptome of *U. decumbens* (Salgado et al., 2017), our species of study.

## Results

### SNP Calling

The GBS library generated a total of 1,183,089,925 raw reads (94% Q20 bases), with an average of 4,151,193 reads per individual. Using the Tassel-GBS pipeline modified to polyploid, alignment with the *S. viridis* genome enabled the identification of 58,370 SNP markers. The output of the Tassel-GBS pipeline was used as the input for the SuperMASSA software, and 9,345 SNP markers with allele dosage were selected with a minimum overall depth of 25 reads. Afterward, markers with up to 25% missing data and non-segregating markers were removed, resulting in 4,240 SNP markers that were used in the subsequent steps.

**FIGURE 1:**
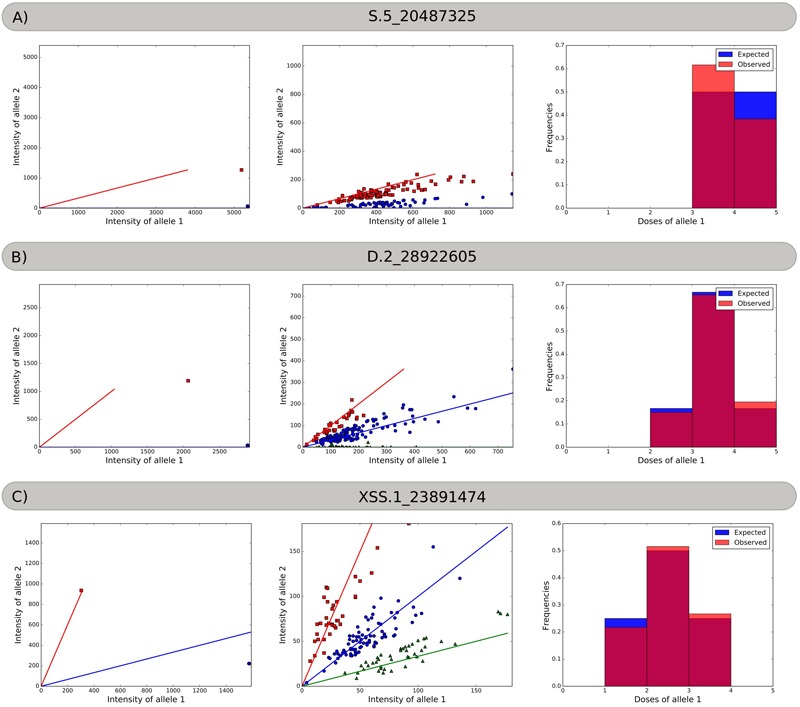
Allele dosage in the parents and progeny, and the frequency histograms. **(A)** Marker S.5_20487325. Red squares represent the Aaaa parent and offspring, and blue circles represent the aaaa parent and offspring. **(B)** Marker D.2_28922605. Red squares represent the AAaa parent and offspring, blue circles represent the aaaa parent and the Aaaa offspring, and green triangles represent the aaaa offspring. **(C)** Marker XSS.1_23891474. Red squares represent AAAa parent and offspring, blue circles represent the Aaaa parent and the AAaa offspring, and green triangles represent the Aaaa offspring.

Using three different SNP markers, Figure 1 exemplifies how the SuperMASSA software uses the ratio of allele count to classify individuals according to their genotypes using a probabilistic graphical model (Serang et al., 2012). The markers were named with the allele dosage configuration, the homology group number, and the chromosomal position of the SNP in the *S. viridis* genome.

GBS sequence data have been submitted to the NCBI Sequence Read Archive (SRA) under accession number SRP148665.

GBS sequence data have been submitted to the NCBI Sequence Read Archive (SRA) under accession number SRP092493.

### Genetic Map

**FIGURE 2:**
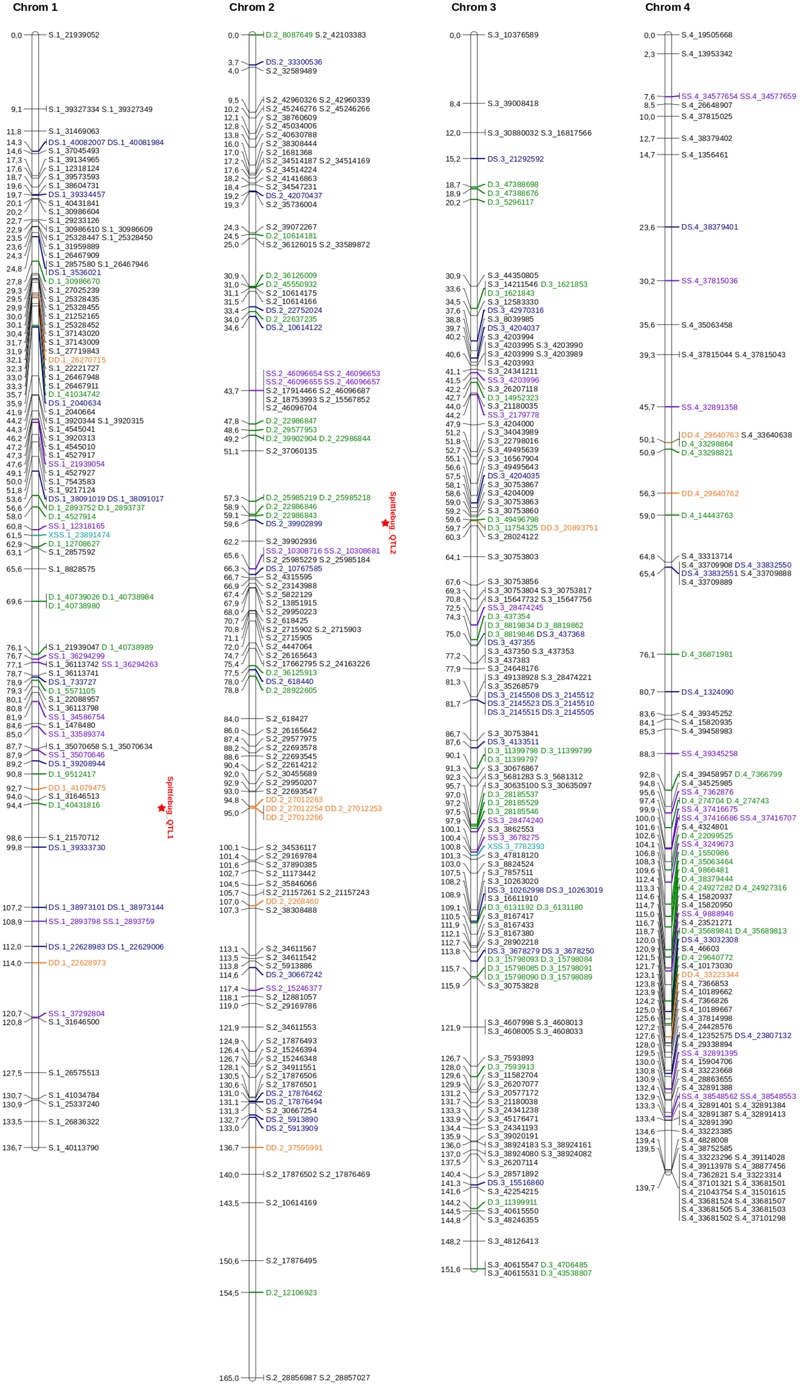
Linkage map for *U. decumbens*: homologous groups from 1 to 4. The genotype configuration of each marker is indicated by the marker name prefix and color. Simplex markers are represented in black; duplex markers are represented in green; double-simplex markers are represented in purple; X-double-simplex markers are represented in light blue; duplex-simplex markers are represented in dark blue and double-duplex markers are represented in orange. QTLs are identified in HG1 and HG2.

**FIGURE 3:**
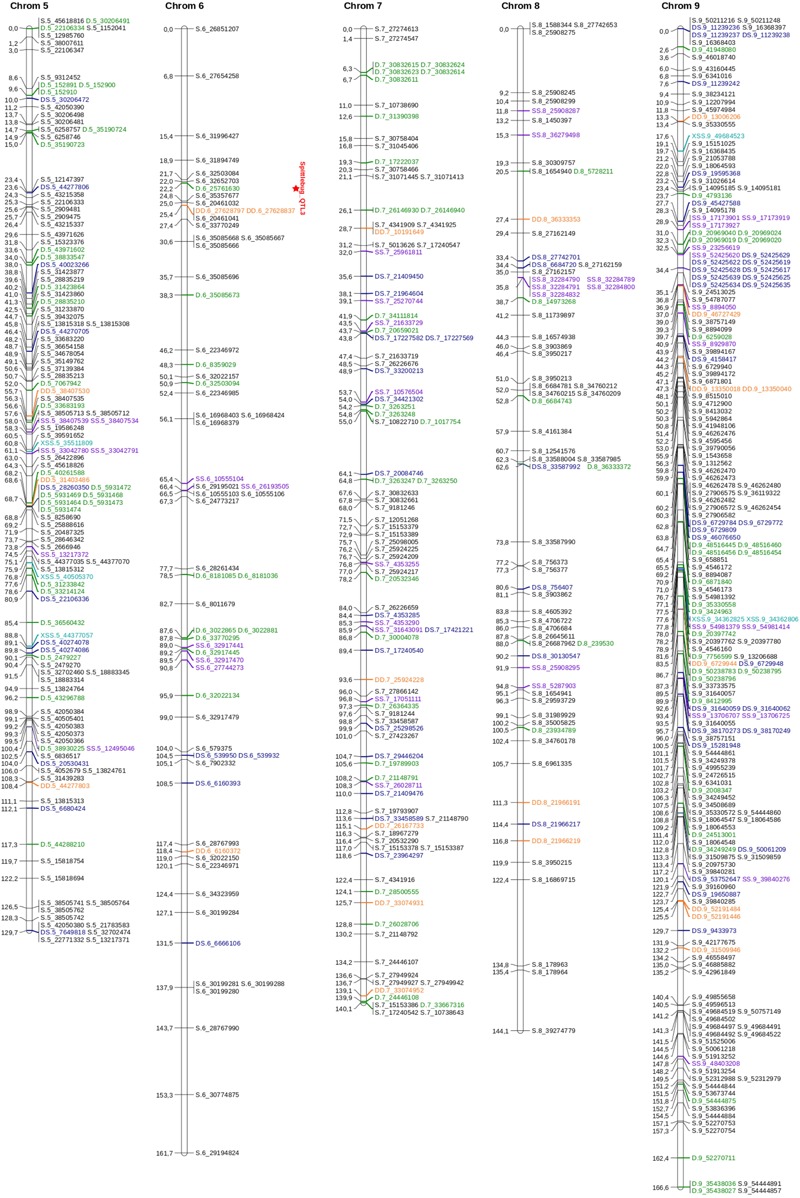
Linkage map for *U. decumbens*: homologous groups from 5 to 9. The genotype configuration of each marker is indicated by the marker name prefix and color. Simplex markers are represented in black; duplex markers are represented in green; double-simplex markers are represented in purple; X-double-simplex markers are represented in light blue; duplex-simplex markers are represented in dark blue and double-duplex markers are represented in orange. A QTL is identified in HG6.

An integrated genetic map was built using 217 full sibs obtained from a cross between *U. decumbens* cv. Basilisk (apomictic cultivar) and *U. decumbens* D24/27 (sexual accession) (Figure 2, 3). Of the 4,240 markers used for linkage analysis, 1,515 showed significance of the χ^2^ goodness-of-fit statistic, and 1,000 were placed in the linkage map (Table 1).

**Table 1:** The number of GBS-based markers after significance testing, the number of mapped markers within each homology group (HG), and the length, marker density and largest distance between adjacent markers in each HG (cM) in the genetic map.

HG	No. GBS-based markers^∗^	No. mapped markers	Length of HG (cM)	Marker density (cM)	Largest distance between adjacent markers (cM)
1	132	100	136.74	1.37	9.1
2	181	121	164.95	1.36	10.5
3	219	138	151.56	1.10	10.7
4	169	101	139.70	1.38	10.7
5	183	123	129.70	1.05	8.4
6	100	63	161.72	2.57	10.4
7	154	99	140.08	1.41	9.1
8	82	68	144.07	2.12	12.4
9	295	187	166.57	0.89	5.2
Total	1,515	1,000	1,335.09	–	–

*^∗^Markers were selected if the χ^*2*^ goodness-of-fit statistic was higher than 0.001 for simplex and 0.01 for higher segregations.*

The markers were distributed throughout nine homology groups (HGs), with each HG therefore reflecting four chromosomes in two parents. The consensus map had a cumulative length of 1,335.09 cM and an average marker density of 1.33 cM. The length of each group ranged from 129.70 cM (HG5) to 166.57 cM (HG9), with an average of 148 cM (Table 1 and Supplementary Table S1). Homology group 9 showed the highest average density and the highest number of mapped markers (Table 1 and Figure 3).

Supplementary Figures S1, S2 and S3 show the parental genetic maps represented by the homology groups. The homology groups on the D24/27 genetic map (female parental) ranged from 116.8 cM (HG8) to 151.8 cM (HG9) in size, with a cumulative map length of 1,208.33 cM and an average marker density of 3.20 cM. The homology groups on the D62 genetic map (male parental) ranged from 129.7 cM (HG5) to 166.57 cM (HG9) in size, with a cumulative map length of 1,335.14 cM and an average marker density of 1.56 cM.

**FIGURE 4:**
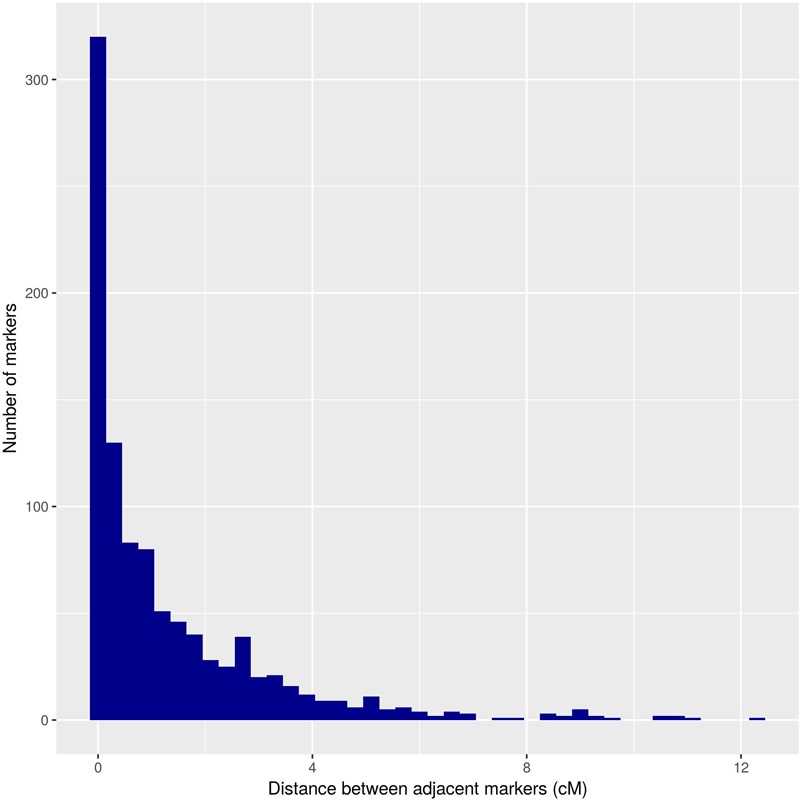
Distribution of the distance between adjacent markers on the *U. decumbens* consensus genetic map.

The distances between adjacent markers on the consensus genetic map were plotted as a histogram, which revealed that the majority of markers were within 1 cM of each other (Figure 4). The two largest distances between adjacent markers were located in HG8 and had lengths of 11.2 cM and 12.4 cM (Figure 3 and Table 1). The largest distance found on the map of the female parental was 25.01 cM (HG6) (Supplementary Figure S2), whereas on the map of the male parental this was 22.04 cM (HG8) (Supplementary Figure S3).

In Figure 2, 3, the possible genotype configuration of each marker is indicated by the marker name prefix and the different colors. For example, markers with the prefix S and the color black represent the simplex configuration (Table 2). Four homologous groups showed almost all configurations (HG1, HG3, HG5 and HG9), and simplex markers (S) were the most frequent. The X-double-simplex (XSS) configuration was less frequent and appeared in only HG3 and HG5 (Figure 2, 3 and Table 2).

**Table 2:** Distribution of SNPs into genotypic classes.

Type	Parent 1	Parent 2	P1 dosage	P2 dosage	SNP initial number	Mapped SNP
Null (N)	AAAA	BBBB	4	0	8	0
Simplex (S)	AAAA	AAAB	4	3	1,332	591
Simplex (S)	ABBB	BBBB	1	0		
Triplex (T)	AAAA	ABBB	4	1	126	0
Triplex (T)	AAAB	BBBB	3	0		
Duplex (D)	AAAA	AABB	4	2	720	172
Duplex (D)	AABB	BBBB	2	0		
Double-simplex (SS)	AAAB	AAAB	3	3	1,013	78
Double-simplex (SS)	ABBB	ABBB	1	1		
X-double-simplex (XSS)	AAAB	ABBB	3	1	65	8
Simplex-duplex (SD)	AAAB	AABB	3	2	11	0
Duplex-simplex (DS)	AABB	ABBB	2	1	773	116
Double-duplex (DD)	AABB	AABB	2	2	192	35
Total					4,240	1,000

### Phenotypic Analysis and QTL Mapping

The selected VCOV matrix for G_a_ based on AIC was CS-Het and/or US, both structures account for genetic correlation and heterogeneous variance across years. Considering the SIC, the selected VCOV matrix for G_a_ was CS, in which the genetic correlation and homogeneous variance across years are considered, in addition to requiring estimation of a lower number of parameters than the previous VCOV matrices. As the difference in SIC values between CS and CS-Het/US (18880.37–18877.39 = 2.98) was greater than the respective AIC difference (18848.03–18845.13 = 2.9), the CS matrix was used for G_a_ (Table 3). The assumption of normality of the residuals can be visualized in Supplementary Figure S4.

**Table 3:** Values of AIC and SIC for G_a_ matrix, considering different VCOV structures.

G_a_ Matrix	Df	AIC	SIC	logREML	
ID	4	18868.02	18891.51	-9430.010	
DIAG	5	18866.02	18895.41	-9428.021	
CS	5	18848.03	**18877.39**	-9419.014	Selected
CS-Het	6	**18845.13**	18880.37	-9416.565	
FA1	7	18847.13	18888.24	-9416.565	
US	6	**18845.13**	18880.37	-9416.565	

*The lowest values are indicated in bold.*

Predicted genotypic values for spittlebug (*N. entreriana*) resistance in progeny and checks, genetic and residual variance components and heritability are shown in Table 4. The predicted mean among the checks ranged from 17.72 (*Urochloa* spp. cv. BRS Ipyporã- highly resistant) to 83.18 (*U. decumbens* cv. Basilisk- highly susceptible) (Table 4). As the variance components for genetic and residual effects were 68.8426 and 382.3238, respectively, the broad sense heritability results in 0.1526 (using $\sigma_{G}^{2}$/($\sigma_{G}^{2}$ + $\sigma_{R}^{2}$)). However, the generalized heritability is more appropriate for unbalanced designs (Cullis et al., 2006), and had value of 0.3730 (Table 4). For balanced designs, the usual broad sense heritability and the generalized heritability coincide (Piepho and Möhring, 2007), which was not our case.

**Table 4:** Summary of the predicted genotypic values for progeny and checks, genetic (${\hat{\sigma }}_{G}^{2}$) and residual (${\hat{\sigma }}_{R}^{2}$) variance and heritability (${\hat{H}}_{C}^{2}$) for spittlebug resistance.

Progeny predicted means			(${\hat{\sigma }}_{G}^{2}$)	(${\hat{\sigma }}_{R}^{2}$)	(${\hat{H}}_{C}^{2}$)
Minimum	Mean	Maximum			
56.16	70.81	85.07	68.86	382.32	0.3730

**Checks**		**Predicted means**		**Classification**	

Basilisk		83.18		Highly susceptible	
Marandu		38.79		Resistant	
Ipyporã		17.72		Highly resistant	
MulatoII		55.96		Intermediary	

Best linear unbiased prediction values were used to perform QTL mapping for spittlebug resistance by applying an IM model to the integrated genetic map. Using this method, three significant QTLs were identified for spittlebug resistance (Table 5) that were located in three homologous groups: HG1, HG2, and HG6. The obtained threshold values for LOD scores using 1,000 permutations ranged from 4.39 to 5.49 and were used to declare the presence of these QTLs. The percentages of phenotypic variation (*R*^2^) explained by the QTLs ranged from 4.66 to 6.24%. The male progenitor of the mapping population, *U. decumbens* D62 (cv. Basilisk), contributed with the resistance alleles for these three QTLs.

**Table 5:** Quantitative trait loci information for the spittlebug resistance trait analyzed in the *U. decumbens* progeny.

Trait	HG	QTL position (cM)	LOD	Variance explained (%)	SIC	Configuration
Nymphal survive	1	95	4.68	4.80	85.33	Duplex (D)
Nymphal survive	2	60	4.39	4.66	93.03	Duplex (D)
Nymphal survive	6	23	5.49	6.24	99.07	Simplex (S)

*HG, homology group; QTL, quantitative trait locus; LODs, logarithm of the odds; SIC, Schwarz Information Criterion.*

In homology group 1, the QTL had a LOD score of 4.68 and explained 4.80% of the phenotypic variation for spittlebug resistance. This LOD score was above the upper 95% LOD permutations threshold of 3.11 (Supplementary Figure S5A). This first QTL was located at position 95 cM (Figure 2). Analyses of different simple genetic models were performed with TetraploidSNPMap to determine the best simple fitting model for the studied trait. For this QTL, the best model was a duplex genotype (AAAA × BBAA), with the allele B present on parent 2 associated with spittlebug resistance. This model had the lowest SIC (SIC = 85.34) compared with the full model (SIC = 102.99) (Table 5).

In homology group 2, the maximum LOD score was 4.39, and this QTL explained 4.66% of the phenotypic variation for spittlebug resistance. The LOD peak was located at position 60 cM (Figure 2), and its score was above the upper 95% LOD permutation threshold of 3.29 (Supplementary Figure S5B). Again, the analysis with the simpler models estimated a duplex genotype (AAAA × BBAA) for this QTL, with the resistance allele present in parent 2. This model had the lowest SIC (SIC = 93.03) in comparison with the full model (SIC = 89.55) (Table 5).

The QTL detected in homology group 6, had the highest LOD score, 5.49, explained 6.24% of the phenotypic variation and was above the 95% LOD permutation upper threshold of 2.79 (Supplementary Figure S5C). The QTL peak was located at 23 cM (Figure 3), and an analysis of different genetic models indicated that an allele explains the phenotypic variation. This QTL is linked to a simplex SNP (AAAA × ABAA), with the B allele present on parent 2 associated with spittlebug resistance. This model had the lowest SIC of 99.07 compared to the full model with SIC of 115.47 (Table 5).

### Search for Candidate Resistance Genes

Using the *S. viridis* genome as a reference, sequence similarity was detected in the regions of the markers adjacent to the mapped QTLs, with homologies for *Arabidopsis thaliana* and *Oryza sativa.* Functional annotation of these sequences showed possible candidate genes involved in the spittlebug resistance response (Supplementary Table S2) that are also present in the *U. decumbens* transcriptome (Salgado et al., 2017).

The first identified QTL, located in HG1, had two adjacent markers, D.1_40431816 and S.1_21570712, identified from the *S. viridis* genome reference. The region of this QTL showed similarity to pathogenesis-related gene 1 (*PR1*), leucine-rich repeat (LRR) family protein and the *WRKY11* gene, which can be involved in the response to insect attacks (Supplementary Table S2).

In HG2, the region of the GBS-based adjacent markers (DS.2_39902899 and S.2_399022936) to QTL2 showed similarity to NBS-LRR and NB-ARC protein domains and with the pentatricopeptide repeat (PPR) superfamily proteins (Supplementary Table S2).

The region of the GBS-based markers (D.6_25761630 and S.6_35357677) adjacent to QTL3 in the HG6 showed similarity to a WRKY DNA-binding protein, a kinase superfamily protein, an F-box family protein, NB-ARC protein domains and kinase superfamily proteins. This QTL region also showed similarity to the thaumatin-like protein superfamily (TLP), which is classified within the pathogenesis-related (PR) protein family, to LRR protein domains and to F-box family proteins (Supplementary Table S2).

## Discussion

The development of GBS technology enables the identification of thousands of SNP markers across the genome of a given species at a relatively low cost. This advance represents an evolution in genetic studies of polyploid species, since a larger part of the genome can be represented, favoring approaches such as the construction of genetic maps. The map reported here was developed using an intraspecific progeny of *U. decumbens* of Embrapa Beef Cattle and includes GBS-based markers with autotetraploid allele dosages, an approach never previously employed in a forage grass species. Although not completely elucidated, some evidence suggests that *U. decumbens* is a segmental allopolyploid (Mendes-Bonato et al., 2001; Worthington et al., 2016), considered to have arisen from hybridization between very closely related species (Stebbins, 1947).

Our molecular markers fit a tetrasomic segregation model (Supplementary Figure S6), probably because autotetraploids, as well as segmental allotetraploids, do not behave as diploids with respect to the mode of inheritance (Bourke et al., 2018). Another explanation is the fact that one of the parents of the mapping population was tetraploidized artificially and therefore behaves as autotetraploid. Therefore, we chose to use all possible SNP configurations to add even more genetic information to the genetic map. Our map provides important genomic information, such as the detection of QTLs involved in spittlebug (*N. entreriana* Berg) resistance, a relevant trait for breeding programs in the *Urochloa* genus.

We used the genome of *S. viridis*, a member of the Poaceae, or grass, family, to align our reads and discover GBS-based markers. *S. viridis* is a species widely used as a model in genetic studies of grasses of the subfamily Panicoideae (Li and Brutnell, 2011), such as *U. decumbens*. *S. viridis* is a phylogenetically related species to *U. decumbens* that currently has a sequenced genome with a better assembly for use in genetic studies such as ours. This alignment was sufficient to identify a relevant number (4,240) of high-quality SNPs with allele dosage estimated. In previous analyses, we tested other reference genomes for our read alignment, but the *S. viridis* genome alignment produced the best results in terms of the number of markers identified (Supplementary Table S3), probably due to genetic proximity with *U. decumbens*.

Worthington et al. (2016) detected 3,912 SNP markers after filtering using the UNEAK (Universal Network-Enabled Analysis Kit) pipeline (Lu et al., 2013), which is similar to the number of markers that we detected using the *S. viridis* genome as reference. In a previous analysis, we tested the UNEAK pipeline for SNP identification in our intraspecific progeny, but this method detected fewer markers (1,210) compared with alignment to the genome reference (4,240). Most likely, the cross between two different species (*U. decumbens* × *U. ruziziensis*) favored the discovery of more SNPs through the UNEAK pipeline by Worthington et al. (2016), in addition to the different methodology used for GBS library construction.

The sequences obtained herein by the GBS libraries are dynamic, allowing the raw data to be reanalyzed as bioinformatics methods evolve (Poland and Rife, 2012), as well as when some tetraploid *Urochloa* spp. genome is sequenced and assembled. We reinforce that our map can help in this process. Therefore, our sequences may be reanalyzed in the future to obtain a higher alignment rate, as well as to identify a larger number of markers.

Previous polyploid genetic studies have been developed with markers scored as presence/absence of alleles, but this method does not make use of all the information available in the SNP at multiple doses. The GBS data obtained here were sufficient in terms of read depth to call allele dosage in 9,345 markers from the *S. viridis* genome using SuperMASSA software (Serang et al., 2012; Pereira et al., 2018) (Supplementary Table S3). This methodology provided the distribution of alleles in the progeny and the relative intensities of each allele, increasing the amount of genetic information obtained (Serang et al., 2012; Garcia et al., 2013; Bargary et al., 2014; Pereira et al., 2018).

After analysis using SuperMASSA software (Serang et al., 2012) and subsequent filtering steps, 4,240 SNPs were obtained representing all possible configurations for an autotetraploid species. Approximately 55% (2,345) of the SNP markers identified from the *S. viridis* genome alignment followed the parental genotype configurations of simplex (AAAA × AAAB/ABBB × BBBB) and double-simplex (AAAB × AAAB/ABBB × ABBB). Slightly less than 1% of the identified markers followed the nulliplex (AAAA × BBBB) and simplex-duplex (AAAB × AABB) configurations (Table 2). These results were satisfactory and relevant for genetic studies with a tetraploid species, but a GBS library construction methodology with more than one restriction enzyme might provide a larger read depth and, accordingly, increase the number of markers detected.

The 4,240 high-quality SNP markers were inserted in TetraploidSNPMap software (Hackett et al., 2017), and 2,725 were identified with distortion from the expected Mendelian segregation ratio. Although numerous studies have shown that markers with segregation distortion can increase the density of the map and help improve QTL detection (Hu et al., 2018), the mapping software used does not allow the inclusion of such markers. Segregation distortion is a common phenomenon that can occur due to non-biological factors such as sampling and genotyping error, or due to biological factors such as gametophyte and/or zygotic selection, hybrid incompatibility, deleterious alleles or chromosomal rearrangements (Manrique-Carpintero et al., 2016; Edae et al., 2017). In our study, crossing of a natural segmental allotetraploid male parent with an artificially autotetraploidized female parent may have caused uneven transmission of the alternative alleles due to reduction of pairing affinity and meiotic abnormalities, causing the appearance of a large number of distorted markers. However, more detailed studies are needed to clarify this hypothesis.

The remaining 1,515 SNPs showed significance in the χ^2^ goodness-of-fit statistic, and 1,000 were placed in the linkage map (Table 1). These 1,000 SNP markers were distributed across nine homology groups, with a total length of 1,335.09 cM and an average map density of 1.33 cM (Figure 2, 3 and Table 1), reflecting the molecular source and mapping methodology.

In Worthington et al. (2016) study, genetic maps for an interspecific progeny resulting from a cross between *U. decumbens* × *U. ruziziensis* were built with GBS-based markers, and 1,916 SDA (single-dose allele) markers were distributed across the parental linkage maps. The different mapping method used in our study may have influenced the number of markers linked in the map. However, although our map has a lower marker density, it contains SNP markers with different allele dosages, adding more genetic information to the map and enabling more robust genetic analyses (Garcia et al., 2013). In addition, our genetic map has a higher density of markers than other *Urochloa* genetic maps (Thaikua et al., 2016; Vigna et al., 2016).

In our study, the density of markers per HG on the consensus map ranged from 63 (HG6) to 187 (HG9) (Figure 2, 3 and Table 1). In relation to the map of the parents, the density of polymorphic markers per HG ranged from 20 (HG6) to 78 (HG9) on the female parental map (D24/27) and from 47 (HG8) to 163 (HG9) on the male parental map (D62) (Supplementary Figures S1, S2 and S3). The greater density of markers for certain HGs may correspond to a greater recombination frequency. However, less saturated groups may have fewer SNP in these regions and/or correspond to highly homozygous regions that have lower recombination frequencies (Bai et al., 2016). We also observed a significant difference in the total number of polymorphic markers on the map of each parental: 377 markers for the female parental genetic map and 856 markers for the male parental genetic map. The lower polymorphic marker density in the map of the female parental (D24/27) is not surprising given the artificial tetraploidization process that this genotype was submitted, which could have a directly influence in the genetic variability on this parent.

The largest distance between adjacent markers on the consensus map, 12.4 cM, was observed in homology group eight (Figure 3 and Table 1), one of the groups with the fewest mapped markers. Compared with other linkage map studies, our map has low average marker intervals in all HGs (Figure 2, 3). Nevertheless, substantial intervals are common and even expected using the GBS technique, mainly due to centromeric regions, which are not reached with this methodology (Conson et al., 2018). Additionally, it is possible that the number of hybrids in the mapping population was not sufficient to observe recombination in these intervals (Ma et al., 2014), that these intervals represent regions that were not captured with the methodology used or that the intervals represent distorted portions of the genome. One possible method to fill these distances is to use different combinations of enzymes to enlarge the sequencing pools and thereby enable the capture of markers in other genomic regions. Furthermore, we mapped only markers identified by aligning with the *S. viridis* genome. Therefore, these observed intervals may contain markers that are exclusive to the *U. decumbens* genome and thus cannot be represented with the methodology used. Therefore, advances in the assembly of complex polyploid genomes are expected to enable the use of the full signalgrass genome as a reference for future studies (Margarido and Heckerman, 2015).

Of the 1000 mapped SNPs, 59% (591) followed the simplex parental genotype configuration (Figure 2, 3 and Table 2). This configuration was also the most frequent in other genetic mapping studies (Hackett et al., 2013; da Silva et al., 2017), probably because it is easily scored and analyzed. The least common configuration was X-double-simplex (XSS), followed by double-duplex (DD) (Table 2), which is the most complete configuration because it allows the representation of all types of segregation for autotetraploid species. Overall, the map displays most of the possible SNP configurations, and these data significantly increase the information about each locus and provide several advantages for genetic analysis (Garcia et al., 2013). When building genetic maps for polyploid species, the use of markers with allele dosage information allows the development of robust genetic maps that allow the detection of QTLs (Hackett et al., 2013; da Silva et al., 2017).

Until recently, no intraspecific progeny have been available for *U. decumbens*; therefore, no QTL mapping studies of this species have been performed to date. The identification of genes/loci underlying spittlebug (*N. entreriana*) resistance is critical for forage grass molecular breeding programs because these pests severely damage pastures (Valle et al., 2009). In our study, QTL mapping for spittlebug resistance was performed by applying interval mapping for the tetraploid progeny (Hackett et al., 2017). Although more accurate QTL models are available (such as composite and multiple interval mapping), these models have not been extended to polyploidy and therefore do not use molecular markers with allele dosage information.

For the multi-allelic QTL analysis, we performed a permutation test to obtain the threshold for declaring significant QTLs. This analysis allowed the identification of three significant QTLs which explained between 4.66 and 6.24% of the phenotypic variation (Table 5). The identification of a small number of QTLs with small effects reflects the median heritability found for spittlebug resistance (*H_C_^2^* = 0.37). Spittlebug resistance in forage grasses is probably not genetically complex but likely involves more than a single major resistance gene (Miles et al., 1995). Therefore, values of phenotypic variation and the number of QTLs detected in our analysis may be mainly due the low genetic variability for spittlebug resistance in the analyzed progeny, as the parents do not differ greatly for this trait and are both considered susceptible. Moreover, the methodology used for QTL detection and/or map saturation may have influenced these results.

Although *U. decumbens* is considered a susceptible species to the pasture spittlebug, our results showed that the segregation of different alleles in each tetraploid parent contributed to the observed polymorphisms in the progeny. Therefore, our results can be useful for breeders to identify the alleles from the male parent that contribute to increased values of this phenotypic trait, consequently allowing genotypes that are more resistant to the development and survival of spittlebugs to be found more rapidly.

Quantitative trait loci with greater effects on spittlebug resistance are more efficient for use in breeding programs, but our results represent a great advance since it is the only study about genetic architecture using an *U. decumbens* intraspecific progeny. Therefore, the detection of these QTLs is the first step in the advancement of genomic studies involving spittlebug resistance. The annotation of sequences that originated markers with mapped QTLs is important to identify candidate genes involved in spittlebug resistance in *U. decumbens*. Thus, the regions that gave rise to these QTLs should be more thoroughly evaluated to identify more genetic information for future applications in the marker-assisted selection in signalgrass breeding programs.

To respond to mechanical damage caused by insect attacks, plants possess multiple molecular defense mechanisms (War et al., 2012). Based on an investigative and comparative analysis, the QTL regions of this study showed similarity to genes containing protein domains reported to be involved in defense responses against biotic stresses in plants (Supplementary Table S2).

For spittlebug resistance trait, we can highlight the similarity of the region of the QTL1 with pathogenesis-related gene 1 (*PR1*). PR proteins are induced by the plants as a defense response system in stress conditions such as insect attacks, as the *PR1* gene is involved in biological processes related to defense responses and systemic acquired resistance (Metzler et al., 1991; Van Loon and Van Strien, 1999; Van loon et al., 2006; Sinha et al., 2014). This region also showed similarity to the *WRKY11* gene, which plays a crucial role in cellular defense responses and defense-related gene transcriptional regulation (Jiang et al., 2016) (Supplementary Table S2).

For QTL3, we can highlight the similarity of the region to the adjacent markers with WRKY DNA binding domains. These transcription factors are involved in plant defense responses to biotic stresses (Du and Chen, 2000; Souza et al., 2017). Moreover, F-box and WRKY DNA binding domains have orthologs in *A. thaliana*, with genes that develop a function in plant defense. This QTL region also showed similarity to Thaumatin-like proteins (TLPs), which play a variety of roles, including plant protection against biotic stress (Liu et al., 2010; Mukherjee et al., 2010; Misra et al., 2016).

The regions of the markers adjacent to the mapped QTLs 2 and 3 showed similarity to NBS-LRR protein domains, which are widely distributed in plants and thought to respond to pathogen attacks, including viruses, bacteria, fungi, and even insects (Song et al., 2017; Souza et al., 2017). NBS-LRRs are the most prevalent class of resistance proteins (Gupta et al., 2012). These regions also showed similarity with F-box proteins that control many important biological functions such as pathogen resistance (Lechner et al., 2006), and the PPR superfamily proteins that have been implicated in plant biotic stress responses (Xing et al., 2018). Moreover, the NB-ARC protein domain is a functional ATPase domain containing a nucleotide-binding site that is proposed to regulate plant disease resistance (Van ooijen et al., 2008).

Although we used the *S. viridis* genome as a reference to investigate the marker regions adjacent to the identified QTLs, we selected only genes that were also found in the *U. decumbens* transcriptome (Salgado et al., 2017) to lend greater reliability to our results. Therefore, our results provide a starting point, indicating possible candidate genes involved in spittlebug resistance in signalgrass. However further studies should be conducted to validate these genomic regions as well as the role of the candidate genes in signalgrass and their effects on phenotypic expression.

We can conclude that this recent opportunity to analyze a *U. decumbens* full-sib progeny has opened new genetic molecular perspectives for this and related species. The genetic map presented herein includes important approaches such as the estimation of tetraploid dosage of each molecular SNP and greatly increases genetic information and represents an important evolution for polyploid studies. In addition, the map allowed the identification of genomic regions related to spittlebug (*N. entreriana*) resistance, the main insect that attacks forage grasses, providing new insights about this trait. Furthermore, the molecular data developed here are dynamic and can be applied in future studies, such as genome assembly and other QTL analyses in *U. decumbens*.

## Author Contributions

LC, SB, CdV, JV, FT, and AdS conceived and designed the experiments. RF, LC, SB, CdV, JV, and FT conducted the experiments and collected data. RF, LL, and AG analyzed the data and interpreted the results. RF, LL, and AdS wrote the manuscript. All authors read and approved the manuscript.

## Conflict of Interest Statement

The authors declare that the research was conducted in the absence of any commercial or financial relationships that could be construed as a potential conflict of interest.

## Footnotes

1 http://www.phytozome.net/

2 http://www.phytozome.org/

## Supplementary Material

FIGURE S1 Homology groups 1, 2, and 3 of the D24/27 maternal linkage map and of the D62 paternal linkage map. The genotype configuration of each marker is indicated by the marker name prefix and color. Simplex markers are represented in black; duplex markers are represented in green; double-simplex markers are represented in purple; X-double-simplex markers are represented in light blue; duplex-simplex markers are represented in dark blue and double-duplex markers are represented in orange.

FIGURE S2 Homology groups 4, 5, and 6 of the D24/27 maternal linkage map and of the D62 paternal linkage map. The genotype configuration of each marker is indicated by the marker name prefix and color. Simplex markers are represented in black; duplex markers are represented in green; double-simplex markers are represented in purple; X-double-simplex markers are represented in light blue; duplex-simplex markers are represented in dark blue and double-duplex markers are represented in orange.

FIGURE S3 Homology groups 7, 8, and 9 of the D24/27 maternal linkage map and of the D62 paternal linkage map. The genotype configuration of each marker is indicated by the marker name prefix and color. Simplex markers are represented in black; duplex markers are represented in green; double-simplex markers are represented in purple; X-double-simplex markers are represented in light blue; duplex-simplex markers are represented in dark blue and double-duplex markers are represented in orange.

FIGURE S4 Residual plots.

FIGURE S5 Interval mapping (IM) for spittlebug resistance from the *U. decumbens* mapping population in chromosomes 1 **(A)**, 2 **(B)**, and 6 **(C)**. Dotted lines indicate the LOD thresholds of 90% and 95% obtained after the permutation tests.

FIGURE S6 Theoretical distribution of genotypes in the mapping population and the distribution of individuals assigned to each genotype. The genotype distributions are nearly identical to the theoretical distribution in an F1 population.

TABLE S1 Homology groups with names, positions and type of markers, and information about phases for each parental.

TABLE S2 Functional description of the sequences found in the regions of the markers adjacent to mapped *Urochloa decumbens* QTLs for spittlebug resistance based on similarity to the *Setaria viridis* genome.

TABLE S3 Number of markers identified in the alignment with five reference genomes using the Tassel-GBS pipeline after allele dosage estimation with SuperMASSA software and R data filtration analysis.
