# Corrigendum: Genetic Mapping With Allele Dosage Information in Tetraploid *Urochloa decumbens* (Stapf) R. D. Webster Reveals Insights Into Spittlebug (*Notozulia entreriana* Berg) Resistance

**DOI:** 10.3389/fpls.2019.00855

**Published:** 2019-06-26

**Authors:** Rebecca Caroline Ulbricht Ferreira, Letícia Aparecida de Castro Lara, Lucimara Chiari, Sanzio Carvalho Lima Barrios, Cacilda Borges do Valle, José Raul Valério, Fabrícia Zimermann Vilela Torres, Antonio Augusto Franco Garcia, Anete Pereira de Souza

**Affiliations:** ^1^Center for Molecular Biology and Genetic Engineering, University of Campinas, Campinas, Brazil; ^2^Genetics Department, Escola Superior de Agricultura “Luiz de Queiroz”, University of São Paulo, Piracicaba, Brazil; ^3^Embrapa Beef Cattle, Brazilian Agricultural Research Corporation, Campo Grande, Brazil; ^4^Plant Biology Department, Biology Institute, University of Campinas, Campinas, Brazil

**Keywords:** allele dosage, *Brachiaria*, linkage map, polyploidy, signalgrass, SNP, quantitative traits

In the original article, there were mistakes in the legend for **Figure 2**, **Figure 3**, **Figure S1**, **Figure S2**, and **Figure S3** as published. Some words and representative colors were incorrectly provided. The corrected legends appear below.

**Figure 2**. Linkage map for *U. decumbens*: homologous groups from 1 to 4. The genotype configuration of each marker is indicated by the marker name prefix and color. Simplex markers are represented in black; duplex markers are represented in green; double-simplex markers are represented in purple; X-double-simplex markers are represented in light blue; duplex-simplex markers are represented in dark blue and double-duplex markers are represented in orange. QTLs are identified in HG1 and HG2.

**Figure 3**. Linkage map for *U. decumbens*: homologous groups from 5 to 9. The genotype configuration of each marker is indicated by the marker name prefix and color. Simplex markers are represented in black; duplex markers are represented in green; double-simplex markers are represented in purple; X-double-simplex markers are represented in light blue; duplex-simplex markers are represented in dark blue and double-duplex markers are represented in orange. A QTL is identified in HG6.

**Figure S1**. Homology groups 1, 2, and 3 of the D24/27 maternal linkage map and of the D62 paternal linkage map. The genotype configuration of each marker is indicated by the marker name prefix and color. Simplex markers are represented in black; duplex markers are represented in green; double-simplex markers are represented in purple; X-double-simplex markers are represented in light blue; duplex-simplex markers are represented in dark blue and double-duplex markers are represented in orange.

**Figure S2**. Homology groups 4, 5, and 6 of the D24/27 maternal linkage map and of the D62 paternal linkage map. The genotype configuration of each marker is indicated by the marker name prefix and color. Simplex markers are represented in black; duplex markers are represented in green; double-simplex markers are represented in purple; X-double-simplex markers are represented in light blue; duplex-simplex markers are represented in dark blue and double-duplex markers are represented in orange.

**Figure S3**. Homology groups 7, 8, and 9 of the D24/27 maternal linkage map and of the D62 paternal linkage map. The genotype configuration of each marker is indicated by the marker name prefix and color. Simplex markers are represented in black; duplex markers are represented in green; double-simplex markers are represented in purple; X-double-simplex markers are represented in light blue; duplex-simplex markers are represented in dark blue and double-duplex markers are represented in orange.

Additionally, the accession number for the GBS sequences, deposited in the NCBI, was wrong. A correction has been made to the **Results** section, subsection **SNP Calling**, paragraph three: “GBS sequence data have been submitted to the NCBI Sequence Read Archive (SRA) under accession number SRP148665”.

The authors apologize for these errors and state that they do not change the scientific conclusions of the article in any way. The original article has been updated.

