# Supplementary figures and images for: Genetic Mapping With Allele Dosage Information in Tetraploid Urochloa decumbens (Stapf) R. D. Webster Reveals Insights Into Spittlebug (Notozulia entreriana Berg) Resistance
Source: Front Plant Sci. 2019 Feb 21;10:92. doi: 10.3389/fpls.2019.00092 (PMC6401981; doi:10.3389/fpls.2019.00092)

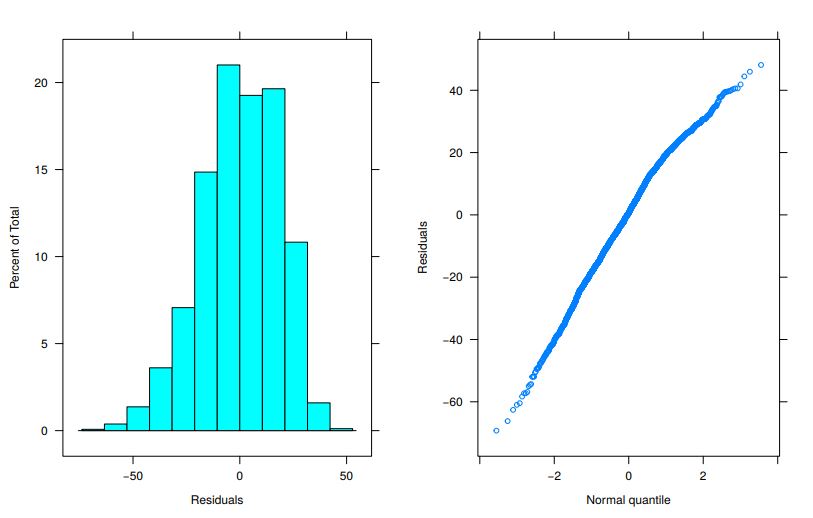

Supplement: FIGURE S4 — Residual plots. [file Image_4.jpg]

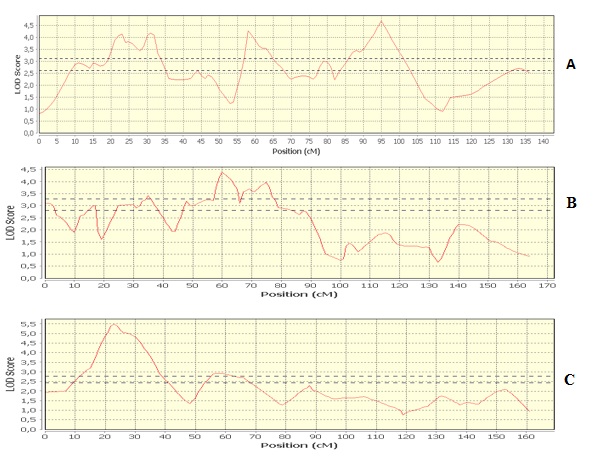

Supplement: FIGURE S5 — Interval mapping (IM) for spittlebug resistance from the U. decumbens mapping population in chromosomes 1 (A), 2 (B), and 6 (C). Dotted lines indicate the LOD thresholds of 90% and 95% obtained after the permutation tests. [file Image_5.jpg]
